# Cultural Psychiatry Teaching: Case Studies from UK Training Programmes

**DOI:** 10.1192/bjo.2026.11338

**Published:** 2026-06-30

**Authors:** Rosalyn Buckland, Megan Parsons, Fabida Aria, Subodh Dave

**Affiliations:** 1 West London NHS Trust, London, United Kingdom; 2 Birmingham & Solihull NHS Trust, Birmingham, United Kingdom; 3 Birmingham & Solihull NHS Trust, London, United Kingdom; 4 Royal College of Psychiatrists, London, United Kingdom

## Abstract

**Aims:**

Cultural psychiatry explores the impact of social and cultural context on mental health and illness. Given new urgency by global anti-racism campaigns, cultural psychiatry teaching has been increasingly prioritised in recent years. However, there is currently little evidence on how cultural psychiatry training is being implemented in practice. By comparing different approaches, this research provides guidance on the benefits and limitations of different formats for teaching cultural psychiatry.

**Methods:**

Four case studies of UK teaching programmes are compared, selected to reflect national variation and diversity of approach. The case studies differ in audience, form andstructure; on whether they were online or in-person; on whether they were mandatory or informal; and in their use of resources.

**Results:**

All groups emphasised the importance of psychological safety when facilitating challenging conversations. Informal sessions were associated with more open conversations; limitations included variable attendance, inadequate organisational time, problems inviting external speakers, and difficulties creating a safe frame. Online spaces were more accessible; but with concerns around engagement, safety and the ability to challenge insensitive comments. Sessions with external speakers brought alternate perspectives, but needed careful management to avoid insensitivity. Consideration was given to whether race and ethnicity were prioritised over other elements of culture such as sexuality, gender, religion and class.

**Conclusion:**

Bringing cultural psychiatry into the curriculum offers the potential to improve both patient care and clinician wellbeing. Lessons learned from these case studies can guide mental health and teaching organisations seeking to implement similar courses.

